# Phase Modulation of Photonic Band Gap Signal

**DOI:** 10.1038/srep28185

**Published:** 2016-06-21

**Authors:** Zhiguo Wang, Mengqin Gao, Abdul Rasheed Mahesar, Yanpeng Zhang

**Affiliations:** 1 Key Laboratory for Physical Electronics and Devices of the Ministry of Education & Shaanxi Key Lab of Information Photonic Technique, Xi’an Jiaotong University, Xi’an 710049, China; 2 School of Science, Xi’an Jiaotong University, Xi’an 710049, China

## Abstract

We first investigate the probe transmission signal (PTS) and the four wave mixing band gap signal (FWM BGS) modulated simultaneously by the relative phase and the nonlinear phase shift in the photonic band gap (PBG) structure. The switch between the absorption enhancement of PTS and the transmission enhancement of PTS with the help of changing the relative phase and the nonlinear phase shift is obtained in inverted Y-type four level atomic system experimentally and theoretically. The corresponding switch in PTS can be used to realize all optical switches. On other hand, the relative phase and the nonlinear phase shift also play the vital role to modulate the intensity of FWM BGS reflected from the PBG structure. And it can be potentially used to realize the optical amplifier.

Optical devices are warmly wanted for the quantum optical information processing as optical components in analogy to the electronic part^1^,^2^. The nonlinear schemes will have very interesting applications in designing novel nonlinear optical devices by selecting compatible driving fields and atomic level schemes. It is well-known that the nonlinear optical effect four wave mixing (FWM)^3^ which can be enhanced or suppressed^4^,^5^,^6^ in an electromagnetically induced transparency (EIT) medium. Additionally, in the EIT^7^,^8^,^9^,^10^ medium, two counter propagating coupling fields can generate the electromagnetically induced grating (EIG)^11^,^12^, which has also been reported on lots of charming studies^13^,^14^,^15^. The EIT medium has the period refractive index and it is essential to generate the photonic band gap (PBG)^16^. The PBG structure controlled by EIG can apply into all optical switch, optical transistor possibly. And an analogy between the modulation of the reflected signal generated by the PBG structure and the amplification function of optical transistor has been demonstrated in the reference^17^.

In this paper, we will research the optical response of rubidium (^85^Rb) atomic vapors driven by a probe field, coupling fields and a dressing field. The transmitted signal and the reflected signal from the photonic band gap structure, which are modulated by the relative phase and the nonlinear phase shift, in EIT based inverted Y-type four level atomic system will be demonstrated experimentally and theoretically for the first time. Through scanning the frequency detunings of the probe and dressing fields, we will show how to obtain the switch between the absorption enhancement of probe transmission signal (PTS) and the transmission enhancement of PTS with the modulation of the relative phase and the nonlinear phase shift. Also the changes of the four wave mixing band gap signal (FWM BGS) (reflected signal from photonic band gap structure) caused by the relative phase and the nonlinear phase shift will be demonstrated in our work. This scheme can provide the new ways in the realization of the all optical switch and optical amplifier.

## Result

**Figure 1:**
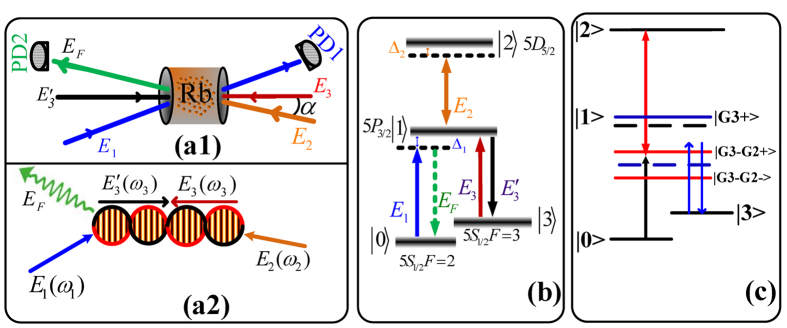
(**a1**) Spatial beams alignment for our experiment. (**a2**) Schematic of an electromagnetically induced grating induced by *E*_*3*_ and 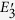. (**b**) Energy-level diagram for the Inverted-Y configuration in ^85^Rb atoms. (**c**) The double dressed energy level schematic diagrams.

In our research, the experiment was implemented in a rubidium atomic vapor cell of ^85^Rb, in which the relevant ^85^Rb energy levels *5S*_1/2_(*F* = *2*) (|0〉), *5S*_*1/2*_(*F* = *3*) (|3〉), *5P*_*3/2*_ (|1〉) and 5*D*_*5/2*_ (|2〉) form an inverted-Y energy system as displayed in Fig. 1(b). As illustrated detailedly, the transition *5S*_*1/2*_ (*F* = *2*) (|0〉) to *5P*_*3/2*_ (|*1*〉) connected by probe *E*_*1*_. The dressing field laser beam *E*_*2*_ connects an upper transition *5P*_*3/2*_ (|1〉) to 5*D*_*5/2*_ (|2〉). And then a pair of coupling laser beams *E*_*3*_ and 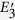 drive the transition *5S*_*1/2*_(*F* = *3*) (|3〉) to *5P*_*3/2*_ (|1〉. The laser beams are aligned spatially as shown in Fig. 1(a1). The weak probe *E*_*1*_ (wavelength of 780.235 nm, frequency *ω*_*1*_ and wave vector *k*_*1*_) propagates in the same direction of 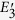 with a small angle through the ^85^Rb vapors. And the coupling field *E*_*3*_ (wavelength of 780.238 nm, *ω*_*3*_, *k*_*3*_) and 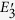 (wavelength of 780.238 nm, *ω*_*3*_, 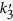) propagate through ^85^Rb vapor in the opposite direction. The dressing field *E*_*2*_ (wavelength of 775.978 nm, *ω*_*2*_, *k*_*2*_) propagates in the same direction of *E*_*3*_ with a small angle *α* between them. In Fig. 1(a2), we can observe a standing wave 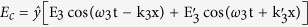 in our system, i.e., electromagnetically induced grating which is generated by the coupling field *E*_*3*_ and 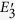 propagating through ^85^Rb vapor in opposite direction. Furthermore electromagnetically induced grating will lead to a PBG structure. The corresponding reflection signal FWM BGS and the transmission signal in the PBG structure can be detected by PD2 and PD1. In addition, because of the small angle between *E*_*1*_ and 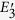, the geometry not only provides a convenient spatial separation of the applied laser and generated signal beams also satisfies the phase-matching (*k*_*F*_ = *k*_*1*_ + *k*_*3*_ − 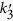). Thus we can probe the generated FWM BGS with highly accuracy^18^. In our research, by changing the value of *α* between the dressing field *E*_2_ and the coupling field *E*_3_, the reflection signal FWM BGS and PTS will be modulated. Figure 1(c) illustrates the dressed state picture used in our system. First, due to the dressing effect of *E*_3_, the level 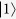 will be split into two dressed states 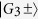 depending on ∆_3_ and |G_3_|^2^. The two dressed states 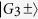 have the eigenvalues 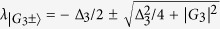. When the probe reaches two-photon resonance Δ_1_ − Δ_3_ = 0, absorption will be suppressed, i.e. the PTS becomes strong. At the same time, the FWM BGS will be suppressed correspondingly. Thus, we define Δ_1_ − Δ_3_ = 0 as the suppression condition. When *E*_*2*_ is turn on, 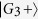 is further split into two dressed states 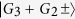 due to the second level dressing effect of *E*_*2*_. The two dressed states 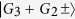 have the eigenvalues 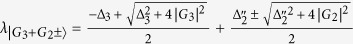 with 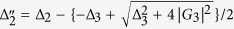. The same way 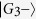 is further dressed into two second level dressed states 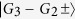, the eigenvalues of which are 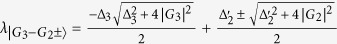, where 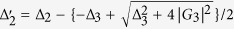.

According to the Liouville pathway^19^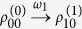, we can get the equation of the first-order density matrix element
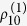
as


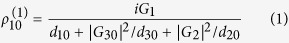


in which 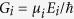
is the Rabi frequency with transition dipole moment*μ*_*i*_, 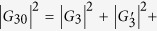

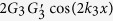
, 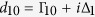, 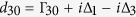, 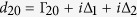, frequency detuning 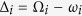 (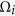 is the resonance frequency of the transition driven by *E*_*i*_) and Γ_*ij*_ is transverse relaxation rate between 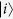 and 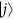.

According to the pathway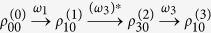, the third-order matrix element 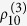
can be obtained as follows


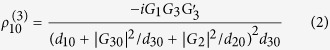


Through the relation 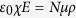, where *ε*_0_, *N* are the dielectric constant and atom density, respectively, we can get the formulations of the linear and nonlinear susceptibilities as follows:


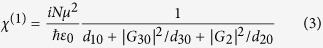



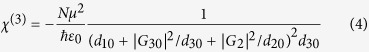


To consider the propagation effect, we introduce an additional phase factor 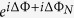 into the dressing term 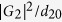. The relative phase 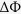 is related to the orientations of induced dipole moments *μ*_1_ and *μ*_2_^20^, which can be manipulated by means of altering the incident angle of *E*_2_. And 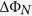 is the nonlinear phase shift induced by *E*_2_^21^, which is proportional to the *n*_2_*I*_2_, where 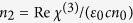 is the nonlinear Kerr coefficient and *I*_*2*_ is the intensity of *E*_*2*_, and can be manipulated by corresponding laser frequency detuning and Rabi frequency. Thus Eqs (1, 2, 3, 4) can be modified as follows:


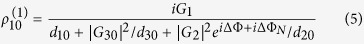



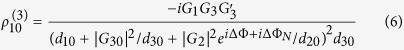



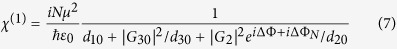



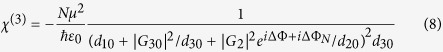


Next we show the nonlinear coupled wave equations in order to estimate the probe transmission signal and the reflection signal,


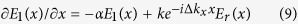



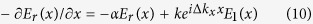


where *E*_*1*_(*x*) and *E*_*r*_(*x*) stand for the probe transmission signal and reflection signal (i.e. *E*_***F***_ in Fig. 1), respectively. 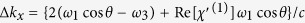 is the phase mismatch magnitude, where *θ* is the angle between probe *E*_1_ and 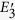. 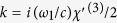 is the gain because of the nonlinear susceptibility and 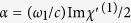 is the attenuation of the field because of the absorption of the medium, in which 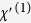, 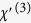 are the zero order coefficients from Fourier expansion of 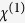,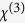, respectively. If length of the sample in x direction is *d*_*x*_, by solving above equations, the reflection signal (*R*) and the probe transmission signal (*T*) are given as


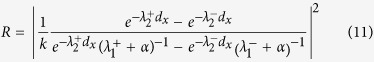



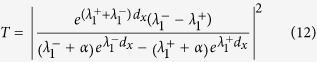


where 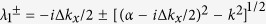,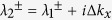.

**Figure 2:**
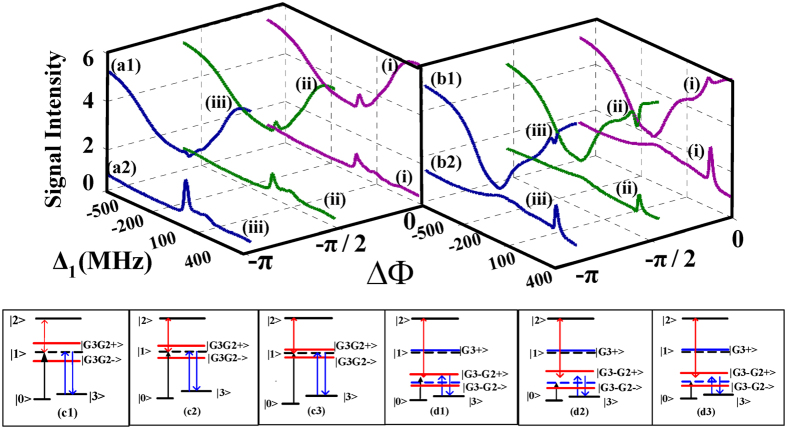
Measured (**a1**) probe transmission signal (PTS), (**a2**) four wave mixing band gap signal (FWM BGS) versus Δ_1_ at different discrete 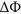 with Δ_3_ = − Δ_2_ = 40 MHz (small detuning); (**b1**) PTS, (**b2**) FWM BGS versus Δ_1_ at different discrete 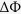 with Δ_3_ = −Δ_2_ = 400 MHz (large detuning). (**c**) Dressed state pictures with (**c1**–**c3**) corresponding to curves (i)–(iii) in (**a1**,**a2**), respectively. (**d**) Dressed state pictures with (**d1**–**d3**) corresponding to curves (i–iii) in (**b1**,**b2**), respectively.

First, we observe the evolutions of PTS and FWM BGS on the condition of scanning Δ_1_ under three typical relative phase 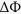 (0, −*π*/2, −*π*) in Fig. 2, respectively. For contrast, we show the modulate effect of 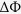 on PTS and FWM BGS under two kinds of conditions i.e., small detuning and large detuning. The PTS and FWM BGS in the case of 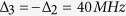
(small detuning) where the nonlinear phase shift related to the frequency detuning, 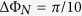, are displayed in Fig. 2(a1,a2), respectively. Under the normal configuration where the relative phase 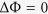 corresponds to the factor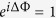, so the dressing terms 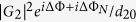 in 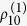 and 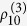
will degenerate to normal ones. Signals under such normal condition are displayed by curves (i) in Fig. 2(a1,a2). The corresponding dressed state pictures are shown in Fig. 2(c1). One can find the transmission enhancement of PTS appears at the three photons resonance position 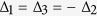. And FWM BGS also appears at 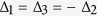 according to the equation of 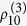. With 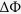 changed to −*π*/2, seeing the curves (ii) in Fig. 2(a1,a2,c2), the transmission enhancement of PTS also appears at the position of 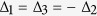. Yet compared with the curve (i) in Fig. 2(a1), the degree of enhancement is weaker. And the intensity of FWM BGS is stronger than the curve (i) in Fig. 2(a2). With 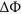 further changed to *π*, seeing the curves (iii) in Fig. 2(a1,a2,c3), the absorption enhancement of PTS appears at 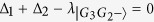 and FWM BGS is stronger than any case we have mentioned (curve (i), curve (ii) in Fig. 2(a2)). Such switch between transmission enhancement of PTS and absorption enhancement of PTS is for the reason that the dressing effect of *E*_*2*_ got modulated by 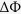 according to the dressing term with a phase factor 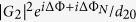 in 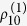 and 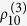. In detail, as shown in Fig. 2(c1,c2), the eigenvalues 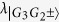 is large on the condition of 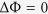 or 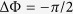 since the dressing effect of *E*_*2*_ is strong. So the number of particle is less at the frequency of two-photon resonance on the dressed level. Thus, the transmission enhancement of PTS appears at the two-photon resonance. In contrast, as shown in Fig. 2(c3), the eigenvalues 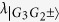 is very small and the dressing effect of *E*_*2*_ is very weak when 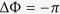, so the probe field will resonance with the dressed state |*G*_*3*_*G*_*2*_*−*〉 i.e., the absorption enhancement of PTS will appears at 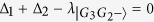. Through comparing the three groups of signals clearly, we can see that the dressing effect of *E*_*2*_ is the strongest when 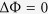, shown as the strongest transmission enhancement of PTS and the lowest intensity of FWM BGS.

Next, let us study the evolutions of PTS and FWM BGS on the condition of 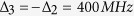 in Fig. 2(b1,b2). Here the nonlinear phase shift 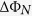 related to the frequency detuning changes to 2/5*π*. With changing 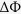 from 0 to −*π*/2 and finally to −*π*, the corresponding PTSs and dressed state pictures are displayed in Fig. 2(b1,d1,d3), respectively. When 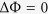, the transmission enhancement of PTS appears at the location of 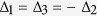. It is because the absorption is decreased at three-photon resonance position. However, the dressing strength of *E*_*2*_ is very weak at this point. When 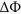 changes to −*π*/2, the dressed state |*G*_*3*_−*G*_*2*_+〉 will be far away from the two-photon resonance position. So the probe will resonance with the dressed state 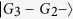 during scanning the probe frequency detuning, thus the absorption enhancement of PTS located at 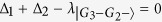. With 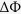 changed to −*π*, the eigenvalues 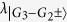 become small since the dressing effect of *E*_*2*_ becomes weak. So the absorption enhancement of PTS at the position of 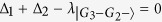 becomes weaker than that in the case of 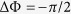. The reason for the conversion is that the dressing effect of *E*_*2*_ changes along with the value of 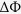 altering according to the dressing term 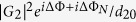 in 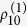. Thus changing 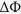 makes PTS change from transmission enhancement to absorption enhancement. The change of FWM BGS with 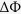 varying is shown in Fig. 2(b2). Through comparing the three signals in Fig. 2(b2), we find that FWM BGS is the lowest in the case of 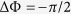 (curve (ii)) and FWM BGS is the highest on the condition of 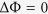
(curve (i)). This can illustrate that the suppression effect of *E*_*2*_ is the strongest when 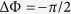 and the weakest when 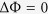. In addition, we can observe the variation in PTS resulting from nonlinear phase shift 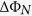 with the fixed 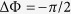 by the curves (ii) in Fig. 2(a1,b1). When we change the Δ_2_ from −40 MHz (curve (ii) in Fig. 2(a1)) to −400 MHz (curve (ii) in Fig. 2(b1)) so as to induce nonlinear phase shift 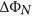 to change from 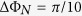 to 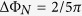, the PTS can switch from the transmission enhancement to absorption enhancement because the dressing effect of *E*_*2*_ also changes with the nonlinear phase shift 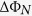.

Compared the two groups of signals which we have mentioned above, it is easy to observe that the transmission enhancement of PTS which can reflect the dressing effect of *E*_*2*_ better in the case of small detuning. But on the condition of large detuning, the dressing effect of *E*_*2*_ can be reflected better by the absorption enhancement of PTS. The reason for the phenomenon is that the nonlinear phase shift 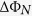 is changing with the frequency detuning of laser beam, which can adjust the distribution of the dressed states. So we can conclude that whether the absorption enhancement or the transmission enhancement of PTS is determined by the nonlinear phase shift 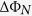 and the relative phase 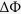.

**Figure 3:**
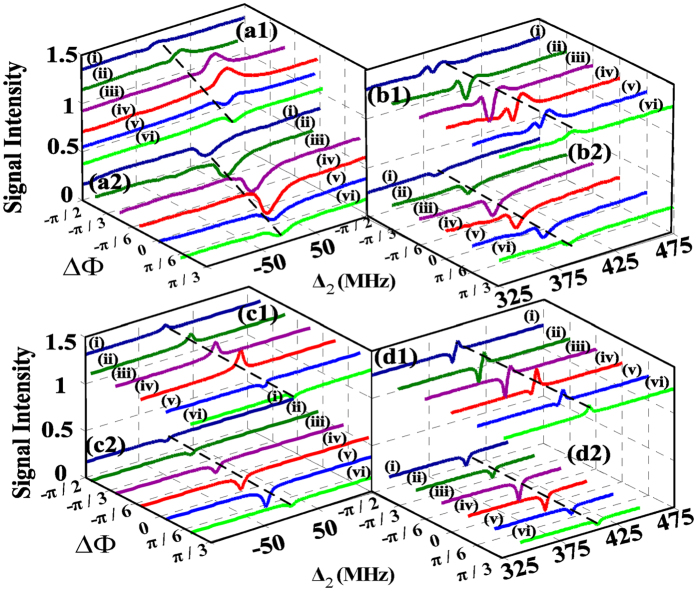
Measured (a1) probe transmission signal (PTS), (a2) four wave mixing band gap signal (FWM BGS) versus Δ_2_ at different discrete values of 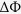 such as −*π*/2 (curve i), −*π*/3 (curve ii), −*π*/6 (curve iii), 0 (curve iv), *π*/6 (curve v), and *π*/3 (curve vi) with Δ_1_ = Δ_3_ = 0 MHz; Measured (b1) PTS, (b2) FWM BGS versus Δ_2_ at different discrete values of 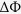 with Δ_1_ = Δ_3_ = − 400 MHz. (c1,c2) are the theoretical calculations of (a1,a2), respectively. (d1,d2) are the theoretical calculations of (b1,b2), respectively.

Further, we concentrate on the variations of the measured signals by setting different fixed values of 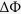
(−*π*/2, −*π*/3, −*π*/6, 0, *π*/6, *π*/3) in the case of scanning Δ_2_ with different nonlinear phase shift 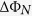
(*π*/10, *π*/2) as depicted in Fig. 3. First, the corresponding PTS and FWM BGS on the condition of 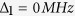 (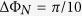) are shown in Fig. 3(a1,a2) respectively. The transmission enhancement of PTS can be switched to the absorption enhancement of PTS gradually along with 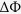 changing from −*π*/2 (curve(i)) to *π*/3 (curve(vi)) due to the dressing term 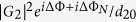 in 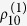. In this process, we find the strongest transmission of PTS appears at 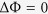 (curve (iv)). In Fig. 3(a2), FWM BGSs are suppressed at the location of 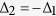 and the intensity of the suppression changes with the relative phase 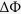 varying according to the dressing term 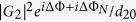 in 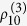. During this process, the strongest intensity of suppression of FWM BGS appears at 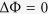 (curve (iv)). Through comparing Fig. 3(a1,a2), we find that the strongest transmission enhancement of PTS and the strongest suppression of FWM BGS appear at the same relative phase 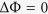. Especially, the calculated PTSs, FWM BGSs according to the equations of R, T as shown in Eq. (11) and Eq. (12) are displayed separately in Fig. 3(c1,c2). Such theoretical calculations confirm our experimental analysis stated above.

Next, Fig. 3(b1,b2) show the large detuning (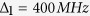) case where the value of nonlinear phase shift 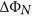 changes to *π*/2. Through scanning dressing field frequency detuning Δ_2_, the absorption enhancement of PTS switches to transmission enhancement of PTS gradually along with 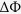 changing from −*π*/2 to *π* in Fig. 3(b1). On the curve (i) where 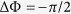, we find that the transmission enhancement of PTS appears at 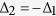 and the absorption enhancement of PTS locates at 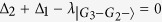. With 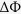 changed to −*π*/6, seeing the curve (iii), the transmission enhancement of PTS and absorption enhancement of PTS both become strong because of the more powerful dressing effect of *E*_*2*_ caused by 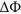. With 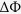 changed to 0, seeing the curve (iv), the absorption enhancement of PTS and the transmission enhancement of PTS both become weak because of the modulate effect of the relative phase 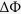. When 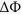 changes to *π*/3 (curve (vi)) finally, only the transmission enhancement of PTS appears at the location of two-photon resonance due to the more feeble dressing effect of *E*_*2*_. Differently in this process, we find the strongest absorption enhancement of PTS appears at 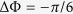 (curve (iii)). It can be also observed that the strongest absorption enhancement of PTS and the strongest suppression of FWM BGS appear at the same relative phase. The phenomena are because that the dressing effect of *E*_*2*_ is various with the relative phase 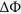 changing according to the dressing term 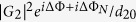 in 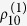 and 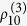. On the condition of 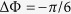 (curve (iii)), the dressing effect of *E*_*2*_ is strongest. In addition, let us observe the variation in PTS resulting from nonlinear phase shift 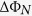 with the fixed 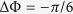 (curve (iii)) in Fig. 3(a1,b1). When we change the detuning Δ_1_ from 0 MHz (Fig. 3(a1)) to 400 MHz (Fig. 3(b1)), corresponding to the nonlinear phase shift 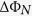 changing from *π*/10 to 2/5*π*, the PTS can switch from the transmission enhancement of PTS to absorption enhancement of PTS because the dressing effect of *E*_*2*_ also changes with the nonlinear phase shift 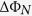. The calculated PTSs, FWM BGSs are displayed separately in Fig. 3(d1,d2). Such theoretical calculations from the equations of R, T confirm our experimental results Fig. 3(b1,b2) very well.

According to the two groups of results, when remaining the nonlinear phase shift 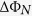
unchanged, PTS can be switched from the transmission enhancement to the absorption enhancement gradually along with the relative phase 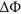. Also PTS can be changed from the transmission enhancement to the absorption enhancement with the nonlinear phase shift 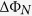 varying on the condition of keeping the relative phase 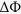 unchanged. So we can modulate the PTS from the transmission enhancement to the absorption enhancement by employing the nonlinear phase shift 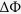 and the relative phase 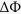. The switching between the absorption enhancement of PTS and the transmission enhancement of PTS can be used to realize all optical switches. So with the help of the nonlinear phase shift 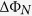 and the relative phase 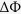, the all optical switch can be more flexible. On other hand, the nonlinear phase shift 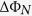 and relative phase 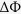 play the role to modulate the intensity of FWM BGS. And it can be potentially used to realize the optical amplifier.

**Figure 4:**
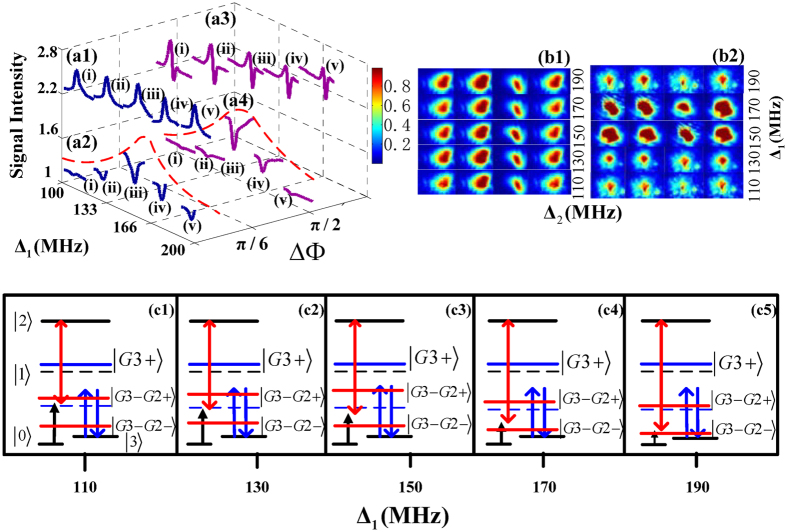
Measured (a1) probe transmission signal (PTS), (a2) four wave mixing band gap signal (FWM BGS) versus Δ_2_ at five different discrete values of Δ_1_ such as 110 MHz (curve i), 130 MHz (curve ii), 150 MHz (curve iii), 170 MHz (curve iv) and 190 MHz (curve v) with Δ_3_ = 150 MHz and 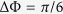. Measured (a3) PTS, (a4) FWM BGS versus Δ_2_ at five different discrete values of Δ_1_ with Δ_3_ = 150 MHz and 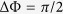. (b1,b2) Measured images of the PTS and FWM BGS versus Δ_2_ and Δ_1_ with Δ_3_ = 150 MHz and 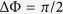, related to (a3,a4), respectively. (c1–c5) dressed state pictures in the case of 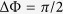
corresponding to curves (i–v) in (a3,a4), respectively.

Finally, we study the switch of PTS and FWM BGS controlled by the nonlinear phase shift 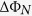
(−3/10, −2*π*/5, −3*π*/5, −4*π*/5, −*π*) in the case of scanning Δ_2_ as depicted in Fig. 4. Figure 4(a1–a4) separately present the measured signals at two specified relative phases 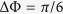 and 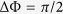. In the PTS shown as in Fig. 4(a1,a3), Peaks higher than baselines are the transmission enhancement of PTS induced by the second level dressing effect of *E*_2_, which appear at 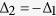 according to the dressing term 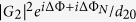 in 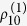. Dips lower than baselines are the absorption enhancement of PTS caused by the dressing effect of *E*_*2*_. When 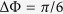, the curves at all positions generally behave the transmission enhancement of PTS in Fig. 4(a1). But when the relative phase changes to 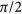 in Fig. 4(a3), we can find the transmission enhancement of PTS appears at the position of 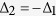 and the absorption enhancement of PTS locates at 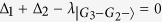 due to the modulation effect of the relative phase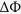. The reason for the phenomenon can seek from the dressed-state pictures in Fig. 4(c). When scanning the dressing frequency detuning Δ_2_, the probe field *E*_1_ resonates with the dressed state 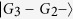 at the location of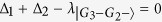, and it also reaches two-photon resonation with the dressing field *E*_2_ at 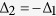. In the following, we can observe the variation of PTS when changing the probe detuning Δ_1_ with the fixed relative phase 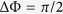 in Fig. 4(a3). With the detuning Δ_1_ changing from 110 MHz (curve (i)) to 190 MHz (curve(v)), the nonlinear phase shift changes from −3*π*/10 to −*π*. As shown by the dressed pictures in Fig. 4(c), the distance between the probe field and the state |*G*_*3*_−〉 (dash line) becomes from short to long and the distance between the probe field and the dressed state 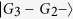 gets from long to short due to the modulation effect of the nonlinear phase shift 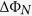. So the transmission enhancement of PTS gradually switches to absorption enhancement of PTS. In addition, images of PTS when scanning Δ_2_ on the condition of 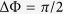 at different Δ_1_ are shown in Fig. 4(b1), which are arranged from bottom to top, corresponding to sub curves from left to right in Fig. 4(a3). We can see the intensity of image is enhanced at 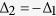 (the second panel from left) and decreased at the position of 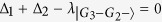 (the third panel from left) in Fig. 4(b1). For FWM BGS in Fig. 4(a2,a4), the profile consisting of the baselines presents the FWM BGS related to *R* obtained from the reflection of PBG structure. The dip in each sub curve shows that FWM BGS is suppressed at 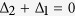
because of the dressing effect of *E*_2_ according to the dressing term 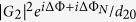 in 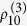. The strongest suppression on FWM BGS appears at 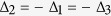. In addition, Fig. 4(b2) provides the images of the FWM BGS for 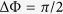 at different Δ_1_ which visually demonstrate the signal intensity evolutions, corresponding to sub curves in Fig. 4(a4). In Fig. 4(b2), the intensity of images is suppressed (the third panel from left) at the location of 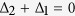 and the intensities of the images in the first and fourth panels from left is consistent with the intensities of baselines in sub curves of Fig. 4(a4). Compared Fig. 4(a2) with Fig. 4(a4), the intensity of suppression on FWM BGS at the same detuning is different when the relative phase is varying. This is because the dressing effect of *E*_2_ is varying with 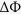 changing. So we can modulate PTS and FWM BGS through changing the relative phase 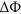 and the nonlinear phase shift 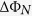.

## Discussion

In summary, the PTS and the FWM BGS manipulated by the relative phase and the nonlinear phase shift in PBG structure are researched experimentally and theoretically. First, when we scan the frequency detuning of probe field, the transmission enhancement of PTS can reflect the dressing effect of field better in the case of small detuning and the absorption enhancement of PTS can reflect the dressing effect of field better on the condition of large detuning. Then in the case of scanning the frequency detuning of dressing field, PTS can be switched from the transmission enhancement to the absorption enhancement gradually along with the relative phase 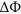 changing. In addition, when we fixed the relative phase, PTS can also be changed from the transmission enhancement to the absorption enhancement with the nonlinear phase shift 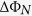 varying. Moreover, the intensity of FWM BGS can also be modulated by the relative phase and the nonlinear phase shift. And the experimental results have been explained carefully through the dressed state pictures in our work. The calculated PTSs, FWM BGSs are also demonstrated separately in our paper. The switch between the absorption enhancement of PTS and the transmission enhancement of PTS can be used to realize all optical switches. And the modulation effect on the intensity of FWM BGS has the potential in realizing the optical amplifier.

## Methods

In the experiment, ***E***_1_, ***E***_2_, ***E***_3_ and ***E***_3_**′** are generated by three external cavity diode lasers (ECDL) with line width of less than or equal to 1 MHz. The coupling laser beams ***E***_3_ and ***E***_3_**′** with a vertical polarization are split from another ECDL. The probe ***E***_1_ is from an ECDL with a horizontal polarization. The dressing laser beam ***E***_2_ with a vertical polarization is from the third ECDL. The power of *E*_*1*_ is the weakest laser beam while the powers of other laser beams are strong. The powers of ***E***_1_, ***E***_3_ and ***E***_3_**′** are 1.9 mW, 16.2 mW and 9.4 mW, respectively. The atomic vapor cell has the typical density of 2 × 10^11^ cm^−3^. We measure the probe transmission signal and the four wave mixing band gap signal in the inverted Y-type four level atomic system. The four wave mixing band gap signals satisfy the phase-matching condition 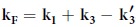.

## Additional Information

**How to cite this article**: Wang, Z. *et al*. Phase Modulation of Photonic Band Gap Signal. *Sci. Rep.* **6**, 28185; doi: 10.1038/srep28185 (2016).
